# Oncostatin M‐Preconditioned Mesenchymal Stem Cells Alleviate Bleomycin‐Induced Pulmonary Fibrosis Through Paracrine Effects of the Hepatocyte Growth Factor

**DOI:** 10.5966/sctm.2016-0054

**Published:** 2016-10-18

**Authors:** Ying‐Wei Lan, Si‐Min Theng, Tsung‐Teng Huang, Kong‐Bung Choo, Chuan‐Mu Chen, Han‐Pin Kuo, Kowit‐Yu Chong

**Affiliations:** ^1^ Graduate Institute of Biomedical Sciences, Division of Biotechnology, College of Medicine, Chang Gung University, Tao‐Yuan, Taiwan, Republic of China; ^2^ Department of Medical Biotechnology and Laboratory Science, College of Medicine, Chang Gung University, Tao‐Yuan, Taiwan, Republic of China; ^3^ Center for Molecular and Clinical Immunology, College of Medicine, Chang Gung University, Tao‐Yuan, Taiwan, Republic of China; ^4^ Department of Preclinical Sciences, Faculty of Medicine and Health Sciences, and Centre for Stem Cell Research, Universiti Tunku Abdul Rahman, Selangor, Malaysia; ^5^ Department of Life Sciences, National Chung Hsing University, Taichung, Taiwan, Republic of China; ^6^ Agricultural Biotechnology Center, National Chung Hsing University, Taichung, Taiwan, Republic of China; ^7^ Rong‐Hsing Translational Medicine Center, National Chung Hsing University, Taichung, Taiwan, Republic of China; ^8^ Department of Thoracic Medicine, Chang Gung Memorial Hospital at Linkou, Tao‐Yuan, Taiwan, Republic of China; ^9^ Department of Medicine, College of Medicine, Chang Gung University, Tao‐Yuan, Taiwan, Republic of China; ^10^ Department of Internal Medicine, Chang Gung Memorial Hospital at Linkou, Tao‐Yuan, Taiwan, Republic of China; ^11^ Molecular Medicine Research Center, College of Medicine, Chang Gung University, Tao‐Yuan, Taiwan, Republic of China

**Keywords:** Oncostatin M, Preconditioning, Stem cells, Bleomycin‐induced pulmonary fibrosis, Hepatocyte growth factor

## Abstract

Mesenchymal stem cells (MSCs) are widely considered for treatment of pulmonary fibrosis based on the anti‐inflammatory, antifibrotic, antiapoptotic, and regenerative properties of the cells. Recently, elevated levels of oncostatin M (OSM) have been reported in the bronchoalveolar lavage fluid of a pulmonary fibrosis animal model and in patients. In this work, we aimed to prolong engrafted MSC survival and to enhance the effectiveness of pulmonary fibrosis transplantation therapy by using OSM‐preconditioned MSCs. OSM‐preconditioned MSCs were shown to overexpress type 2 OSM receptor (gp130/OSMRβ) and exhibited high susceptibility to OSM, resulting in upregulation of the paracrine factor, hepatocyte growth factor (HGF). Moreover, OSM‐preconditioned MSCs enhanced cell proliferation and migration, attenuated transforming growth factor‐β1‐ or OSM‐induced extracellular matrix production in MRC‐5 fibroblasts through paracrine effects. In bleomycin‐induced lung fibrotic mice, transplantation of OSM‐preconditioned MSCs significantly improved pulmonary respiratory functions and downregulated expression of inflammatory factors and fibrotic factors in the lung tissues. Histopathologic examination indicated remarkable amelioration of the lung fibrosis. LacZ‐tagged MSCs were detected in the lung tissues of the OSM‐preconditioned MSC‐treated mice 18 days after post‐transplantation. Taken together, our data further demonstrated that HGF upregulation played an important role in mediating the therapeutic effects of transplanted OSM‐preconditioned MSCs in alleviating lung fibrosis in the mice. Stem Cells Translational Medicine *2017;6:1006–1017*

**Significance Statement**

In this study, the investigation of a cytokine, oncostatin M (OSM), has been extended. The results showed that OSM preconditioning of mesenchymal stem cells (MSCs) could lead to comprehensive modification of gene regulation that enhances cell proliferation and migration and attenuates extracellular matrix production. Hence, transplantation of OSM‐preconditioned MSCs improved pulmonary functions and reduced inflammatory and fibrotic mediators in a bleomycin‐induced pulmonary fibrosis mouse model. The data further suggest that the upregulation of hepatocyte growth factor plays a key role in mediating the therapeutic effects of transplanted OSM‐preconditioned MSCs.

## Introduction

Idiopathic pulmonary fibrosis is a progressive and irreversible lung‐restricted interstitial disease characterized by alveolar epithelial cell injury, inflammatory cell infiltration, decrement of lung volumes, and impaired pulmonary functions that eventually results in death [1, 2]. Different approaches of mesenchymal stem cell (MSC)‐based cell therapy have been reported for multiple lung diseases [3]. MSCs have the ability to specifically target sites of injury, leading to antiapoptotic properties, modulation of immune responses, repair of epithelial tissues, attenuation of extracellular matrix deposition, and modification of the microenvironment at the engraftment sites [4, 5]. In addition to direct cell‐cell interaction, MSCs also act via a paracrine mechanism in secreting soluble factors that account for anti‐inflammatory and antifibrotic effects in vitro and in vivo [6, 7]. However, a major challenge that is limiting the potential of MSC‐based cell therapy is the extensive loss of transplanted cells and low paracrine activities due to exposure to harsh microenvironmental conditions and inflammation [8]. Ex vivo manipulation of MSCs is needed to enhance proliferation, migration, and antiapoptotic capacity and to maximize the paracrine action of MSCs before transplantation to improve the efficacy of MSCs transplantation [9, 10]. Our previous study demonstrated that preconditioning MSCs with sublethal physical stimulation by hypoxia prior to transplantation bestowed higher cytoprotective abilities and exerted better therapeutic effects in bleomycin (BLM)‐induced pulmonary fibrotic mice [11]. In addition, a number of pharmacological preconditioning agents [12, 13, 14] have been shown to increase cell viability of engrafted cells through upregulating multiple prosurvival, antioxidant, or antiapoptotic genes and via enhanced paracrine effects, leading to improved functional recovery in an ischemic disease model.

Oncostatin M (OSM) is a pleiotropic cytokine that belongs to the interleukin (IL)‐6 subfamily that is upregulated in tissues of various airway diseases [15]. OSM has been shown to signal through two receptors: The type 1 receptor is a heterodimer of leukemia inhibitory factor receptor (LIFR) and gp130; the type 2 receptor is a heterodimer of OSM receptor β chain (OSMRβ) and gp130 [16]. However, mouse OSM exclusively binds to the type 2 receptor and exerts multiple physiological functions through activating various signal cascades [16, 17]. In addition, enhancing the responsiveness of the damaged hepatocytes to interact with OSM by upregulating the OSMR significantly inhibited hepatocyte apoptosis and promoted hepatocyte proliferation, leading to active liver regeneration [18].

In this work, we investigated, in a mouse fibrotic lung model, whether OSM preconditioning of MSCs would upregulate expression of OSM receptors to enhance interaction with OSM to promote survival of transplanted cells via enhanced cytoprotection and the mechanism of such events.

## Materials and Methods

### Chemicals

Bleomycin sulfate from *Streptomyces verticillus* was obtained from Sigma‐Aldrich (St. Louis, MO, https://www.sigmaaldrich.com). The c‐Met inhibitors PHA‐665752 and SU‐11274 were purchased from Santa Cruz Biotechnology, Inc. (Dallas, TX, https://www.scbt.com) and Selleckchem (Houston, TX, http://www.selleckchem.com), respectively. Oncostatin M was obtained from R&D Systems (Minneapolis, MN, https://www.rndsystems.com).

### Cell Lines

Mesenchymal stem cells (MSCs) were isolated from bone marrow of C57BL/6 female mice according to the guidelines of the Association for Assessment and Accreditation of Laboratory Animal Care International. C57BL/6 MSCs were also purchased from Thermo Fisher Scientific Life Sciences (Waltham, MA, http://www.thermofisher.com). MSCs stably expressing the LacZ gene (β‐Gal‐MSCs) via a lentiviral system, as previously described [11]. Cell lines were maintained in Dulbecco's modified Eagle's medium/Ham's nutrient mixture F‐12 (DMEM/F12) (Thermo Fisher), supplemented with 10% fetal bovine serum (FBS) (Thermo Fisher), 2 mM l‐glutamine, and 1% penicillin/streptomycin. Human 14‐week male embryonal lung cell line MRC‐5 (BCRC‐60023) was purchased from Bioresource Collection and Research Center (Hsinchu, Taiwan, http://www.bcrc.firdi.org.tw). MRC‐5 cells were maintained in Eagle's Minimal Essential Medium (MEM) (Thermo Fisher), supplemented with 10% FBS and 1% penicillin/streptomycin and were incubated at 37°C in a 5% CO_2_ incubator. Mouse macrophage‐like cell line RAW264.7 (BCRC‐60001) was purchased from Bioresource Collection and Research Center. Cell lines were maintained in DMEM containing 10% heat‐inactivated FBS.

### Oncostatin M Preconditioning

MSCs grown to confluence were treated with indicated concentrations of OSM for 24 hours. Conditioned medium was collected from MSCs that were cultured in the presence or absence of OSM.

### shRNA Transduction

The hepatocyte growth factor (HGF) shRNA clone (TRCN0000336131; GGTAAAGGAGGCAGCTATAAA) targeted at the mouse HGF transcript was purchased from the National RNAi Core Facility, Academia Sinica (Taipei, Taiwan, http://rnai.genmed.sinica.edu.tw). Lentiviral constructs were generated by using three packaging plasmids—pMDLg/pRRE, CMV‐VSVG, and RSV‐Rev—in 293T cells, as described by Chen et al. [19]. MSCs were treated with viral supernatant and polybrene for 24 hours before selection with 0.5 μg/ml puromycin.

### Cell Proliferation Test

MSCs were plated at a density of 1 × 10^4^ cells per well in a 12‐well culture plate and incubated overnight to allow adhesion. The cells were cultured under serum‐starved condition and treated with the indicated concentration OSM for 24 or 48 hours before being detached by trypsinization for cell count.

### Coculture MSCs With Fibroblast

Two different coculture experiments were performed: (1) MSCs and MRC‐5 cells were plated in transwells and six‐well culture plates, respectively, and were cultured overnight. MSCs were then treated with the indicated OSM concentrations for 24 hours. MRC‐5 cells were treated with or without 2.5 ng/ml transforming growth factor (TGF)‐β1 (Sino Biological Inc., Beijing, China, http://www.sinobiological.com) for 24 hours. After removing the medium and washing the cells with phosphate‐buffered saline (PBS), cocultures of OSM‐pretreated MSCs and MRC‐5 cells were conducted in mixed culture medium (1:1 mixture of DMEM/F12 and MEM media) for 24 hours. (2) MRC‐5 cells were seeded in the culture plate in the presence or absence of TGF‐β1 and simultaneously incubated with a mixture of MEM culture media and MSCs or OSM‐MSC‐conditioned medium (1:1) for 24 hours. The MRC‐5 cells were harvested for determination of fibronectin mRNA expression level by quantitative real‐time reverse transcription‐polymerase chain reaction (RT‐qPCR).

### Coculture MSCs With Macrophages

RAW264.7 cells incubated with a mixture of DMEM common culture media and MSCs or OSM‐MSC‐conditioned medium (1:1) and were simultaneously treated with lipopolysaccharides (LPS) (10 ng/ml) for 7 hours. RT‐qPCR was performed to determine the pro‐IL‐1β and IL‐6 mRNA level in the cells.

### Scratch Wound Assays

MRC‐5 cells were plated and maintained for 24 hours to confluence. The confluent monolayers were then scratched with a sterile pipette tip to leave a straight wound. Culture medium was immediately removed, and the cells were rinsed with PBS before adding opti‐MEM medium or MSC‐conditioned medium. Wound areas were monitored, and digitized images were captured with an inverted microscope under a bright field at different time points. Digitized images were analyzed by TScratch software (Zurich, Switzerland, www.cse‐lab.ethz.ch/software.html) [20] in triplicate.

### PHA‐665752 and SU‐11274 Treatment

MRC‐5 cells were seeded and incubated overnight to allow adhesion. MRC‐5 cells were treated with or without 1 μM of PHA‐665752 (Sigma‐Aldrich) or 0.5 μM of SU‐11274 (Selleckchem) for 24 hours. Conditioned medium (CM) from MSCs or OSM‐pretreated MSCs with 2.5 ng/ml TGF‐β1 (Sino Biological) were then used to treat MRC‐5 cells for 24 hours.

### Flow Cytometry Analysis

Flow cytometry was used to confirm the presence of OSMR and gp130 on the cell surface. Detached MSCs were washed twice in staining buffer (PBS containing 1% bovine serum albumin) and incubated with phycoerythrin (PE)‐labeled mouse gp130 at 1:50 dilution (MBL International, Woburn, MA, https://www.mblintl.com) or rat anti‐mouse OSMR (0.4 μg/ml) (MBL International) before incubation with PE‐conjugated goat anti‐rat IgG (1:100) (Beckman Coulter, Brea, CA, https://www.beckmancoulter.com) at 4°C for 30 minutes. After rinsing the cells twice with staining buffer, fluorescence was detected and analyzed by using flow cytometry.

### RNA Isolation and RT‐qPCR

Total RNA was prepared from the cell lines and tissue by using a total RNA mini‐kit (Geneaid, Taipei, Taiwan, http://www.geneaid.com). RNAs were reverse transcribed into cDNAs at 42°C for 60 minutes using Moloney Murine Leukemia Virus Reverse Transcriptase (Thermo Fisher). After the oligo (dT)‐primed reverse transcription reaction, RT‐qPCR was performed by using LightCycler 480 SyberGreen I Master Mix and LightCycler 480 Instrument (Roche, Mannheim, Germany, http://www.roche.com), as has been previously described [21]. Sequences of the mouse gene‐specific primers used and for the human fibronectin are listed in supplemental online Table 1. Relative gene expression was determined by the △△C_t_ method, where C_t_ was the threshold cycle. The relative mRNA levels were normalized to the mRNA level of the reference glyceraldehyde‐3‐phosphate dehydrogenase gene.

### Western Blot Analysis

Western blot analysis was performed, as has been previously described [22]. The antibodies used were anti‐OSMR (MBL International), anti‐HGF (Santa Cruz Biotechnology), and anti‐β‐actin (Millipore, Billerica, MA, http://www.emdmillipore.com) antibodies.

### Determination of Total Collagen Content in Cultured Cells

Total collagen content was evaluated with the Sirius Red/Fast Green Collagen detection kit (Chondrex Inc., Redmond, WA, https://www.chondrex.com) according to the manufacturer’s instructions. Briefly, cultured cells were stained with Sirius Red and Fast Green for collagen and noncollagen proteins, respectively. The dyes were extracted and measured at OD540 for Sirius Red and OD605 for Fast Green. The relative collagen content was calculated and expressed as collagen/total protein.

### Animal Model of BLM‐Induced Lung Fibrosis

Pulmonary fibrosis was induced by intratracheal instillation, as has been previously described [11]. Eight‐week‐old male C57BL/6JNarl mice were purchased from the National Laboratory Animal Center (Taipei, Taiwan, http://www.nlac.narlabs.org.tw). All experimental procedures were approved by the Institutional Animal Care and Use Committee of the University of Chang Gung University. Each C57BL/6JNarl mouse was intratracheally administered 1.5 mg/kg BLM dissolved in 50 μl of sterile PBS on day 0. On day 3, mice were randomly selected for intratracheal injection of MSCs (2 × 10^5^ in 50 μl of PBS), OSM‐preconditioned MSCs (OSM‐MSCs, 2 × 10^5^ in 50 μl of PBS), OSM‐preconditioned HGF knockdown MSCs (OSM‐shHGF‐MSCs, 2 × 10^5^ in 50 μl of PBS), or PBS. On days 4 and 18, after stem cell engraftment, the pulmonary functions were examined, and the mice were sacrificed by an overdose of 2.5% avertin.

### Noninvasive Measurement of Pulmonary Function by Whole‐Body Plethysmography

All mice were placed in the whole‐body plethysmograph (Data Sciences International, Brighton, MN, http://www.datasci.com), as has been described previously [11]. The baseline‐enhanced respiratory pause (Penh) was measured for 3 minutes in each experiment to indicate altered airway function [23].

### Bronchoalveolar Lavages Fluid Collection and Analysis

Lungs were lavaged with 1.5 ml of cold PBS, and the recovery rate of bronchoalveolar lavages fluid (BALF) was approximately 85%. Cells were isolated from the BALF by centrifugation at 300 *g* for 5 minutes at 4°C, and the cell pellets were resuspended in 500 μl of PBS; the obtained BALF samples were analyzed by using a flow‐cytometric hematology system (XT‐2000iV; Sysmex Corporation, Mundelein, IL, https://www.sysmex.com) to classify the number and type of inflammatory cells. The total cells in BALF were counted by a hemocytometer, and cell viability was measured by trypan blue exclusion.

### Lung Water Content Measurement

Lung (lower left lobe) wet weight was determined. Subsequently, the lungs were incubated at 65°C overnight to remove all moisture. The dry weight of lung tissue was then measured, and the percentage of water content in the lung was calculated using the following equation: (wet weight − dry weight)/wet weight × 100%.

### Lung Morphometry

Left lungs were fixed with 10% formalin and embedded in paraffin sections. The sections then stained with hematoxylin and eosin (H&E) and Masson's trichrome staining, according to standard protocols [24]. The severity of lung fibrosis was assessed by measuring the Ashcroft score [25] for quantitative histological analysis of BLM‐induced fibrotic changes. Five random fields within each lung section were observed, and the score of fibrosis ranged from 0 (normal lung) to 8 (total fibrous obliteration of the field) [26].

### β‐Gal Immunohistochemical Staining

Immunohistochemical staining of the paraffin‐fixed sections to evaluate engraftment of transplanted β‐Gal‐MSCs in vivo was performed, as has been previously described [27]. The stained sections were scanned by the HistoFAXS (TissueFAX plus; TissueGnostics, Vienna, Austria, http://www.tissuegnostics.com).

### Total Lung Collagen Measurements

Total lung collagen was determined by using the Sircol Collagen Assay kit (Biocolor Ltd., Belfast, UK, http://www.biocolor.co.uk), according to the manufacturer’s instructions [11].

### Statistical Analysis

Data are represented in vertical scatter dot plots with the mean or are shown in bar graphs displaying mean ± SD. Comparisons between two groups were analyzed with two‐tailed Student’s *t* test. For multiple comparisons, one‐way analysis of variance was used, followed by Dunnett’s post hoc test. All statistical analyses were performed with GraphPad Prism (GraphPad Software, Inc., San Diego, CA, https://www.graphpad.com). For all analyses, a *p* value <.05 was considered statistically significant.

## Results

### Oncostatin M Preconditioning Upregulates the OSM Receptor Complex and HGF Expression in MSCs

**Figure 1:**
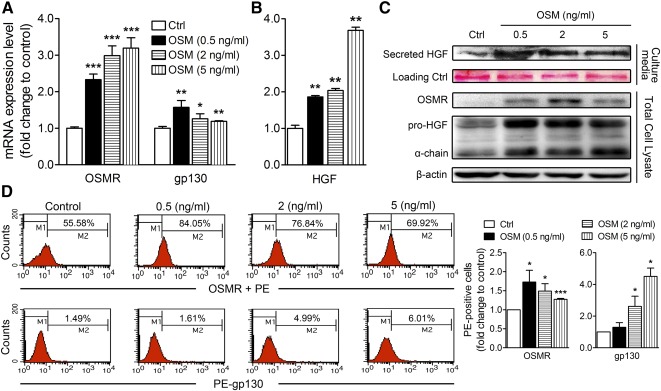
Oncostatin M preconditioning upregulates expression of OSM receptor complex and hepatocyte growth factor in mesenchymal stem cells. Real‐time polymerase chain reaction quantification is shown of the relative expression levels of mRNAs of OSM receptor and gp130 **(A)** and HGF **(B)** in MSCs treated with different dosages of OSM for 24 hours. Values were normalized to glyceraldehyde‐3‐phosphate dehydrogenase and are expressed in relation to the respective control group. **(C):** Western blot analysis of OSMR and HGF in total cell lysates and secreted HGF in culture media. Ponceau S staining of the membrane was presented (row 2) as the loading control. **(D):** Flow cytometry analysis of the percentage of MSCs positive for OSMRβ and gp130. The quantitative histogram on the right shows the data of three independent MSC samples of phycoerythrin‐positive cells. ∗, *p* < .05; ∗∗, *p* < .01; ∗∗∗, *p* < .001. Abbreviations: Ctrl, control; HGF, hepatocyte growth factor; OSM, oncostatin M; OSMR, OSM receptor; PE, phycoerythrin.

A pilot study was first conducted to determine the expression level of OSM receptor (OSMR) complex and downstream factors and to establish the optimal OSM preconditioning concentration. The mRNA levels of both OSMR and HGF showed a gradual increase when MSCs were treated with increasing concentrations of OSM from 0.5 to 5 ng/ml (Figs. 1A, 1B). The mRNA level of gp130 peaked at 0.5 ng/ml OSM exposure (Figs. 1A, 1B). To confirm the specificity of OSM signal transduction, we also examined the IL‐6 family‐related receptors IL‐6R and LIFR, and they showed downregulated expression on treatment with different dosages of OSM (supplemental online Fig. 1A). An increased level in secreted HGF protein was observed at 0.5 ng/ml OSM, but OSM treatment at higher concentrations showed no significant effects that were different from 0.5 ng/ml (Fig. 1C). Hence, the optimal OSM concentration was determined to be 2 ng/ml and was used in subsequent experiments.

To evaluate the percentage of OSM‐sensitive cells in the total MSC population following exposure to different dosages of OSM, we performed flow cytometric analysis. The data showed that the percentage of gp130‐positive MSCs increased in a dose‐dependent manner after 24 hours of treatment at 0.5 to 5 ng/ml. A maximal percentage of OSMR‐positive MSCs was observed at 0.5 ng/ml OSM exposures (Fig. 1D). These results indicated that low‐dose OSM preconditioning was already increasing the expression of OSM receptor complex (OSMR/gp130) and inducing HGF secretion.

### Oncostatin M Preconditioning Modulated Wound Healing, Cell Proliferation, Inflammatory Response, and Extracellular Matrix Remodeling Through Paracrine Hepatocyte Growth Factor

**Figure 2:**
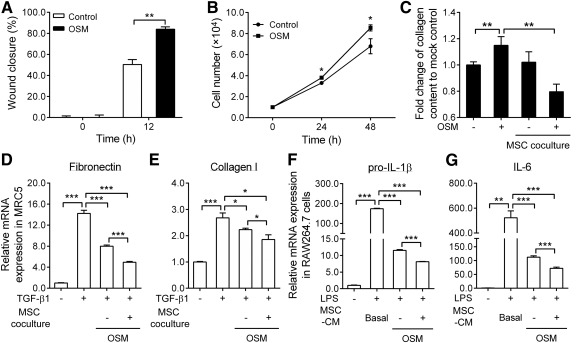
Oncostatin M preconditioning enhances mesenchymal stem cell proliferation, increases wound healing, attenuates inflammatory effects, and decreases extracellular matrix production in fibroblast cells. **(A):** Scratch wound assay of the paracrine effect of OSM‐MSCs on MRC‐5 fibroblast migration. Quantification of wound closure was assessed by TScratch software after 12 hours. **(B):** Proliferation of MSCs under serum‐starved condition and treated with 2 ng/ml of OSM for 24 and 48 hours; cell number was counted by a hemocytometer. **(C):** Inhibitory effects of MSCs on collagen production. Cocultured MSCs (upper chamber) and MRC‐5 (lower chamber) were simultaneously incubated with OSM for 24 hours, followed by measurement of collagen production with Sirius Red/Fast Green Collagen detection kit. **(D, E):** To assess the antifibrotic effects of MSCs, we seeded untreated MSCs or OSM‐MSCs in the upper chamber, and transforming growth factor‐β1‐treated MRC‐5 cells were seeded in the lower chamber in the coculture experiments. After 24‐hour incubation, quantitative real‐time reverse transcription‐polymerase chain reaction (RT‐qPCR) was performed to determine the mRNA levels of fibronectin and collagen type I in the cells. **(F, G):** To assess the anti‐inflammatory effects, we incubated lipopolysaccharides (10 ng/ml)‐treated RAW264.7 cells simultaneously with a mixture of RAW264.7 basal media and OSM‐MSC‐conditioned medium or MSC basal media (Dulbecco's modified Eagle's medium/Ham's nutrient mixture F‐12; control) (1:1) for 7 hours. RT‐qPCR was performed to examine changes in the pro‐interleukin (IL)‐1β and IL‐6 mRNA levels in the cells. Values were normalized to the glyceraldehyde‐3‐phosphate dehydrogenase gene and are expressed in relation to the control group. ∗, *p* < .05; ∗∗, *p* < .01; ∗∗∗, *p* < .001. Abbreviations: CM, conditioned medium; h, hour; IL, interleukin; LPS, lipopolysaccharides; MSC, mesenchymal stem cell; OSM, oncostatin M; TGF, transforming growth factor.

OSM has multiple biological activities, including activation of proliferation in human adipose tissue‐derived MSCs [28], acceleration of wound healing in fibroblasts [29], and regeneration of various tissues [30, 31]. MRC‐5 fibroblast cells were used to examine the chemotactic effects of paracrine factors secreted by OSM‐preconditioned MSCs in accelerating wound healing. Hence, wound healing effects were investigated in MRC‐5 cells cultured in conditioned medium (CM) derived from cultures of MSCs treated without or with OSM. CM from low‐dose (2 ng/ml) OSM treatment significantly promotes wound healing compared with CM from untreated MSCs (Fig. 2A). Therefore, low‐dose OSM (2 ng/ml) was used for subsequent experiments. MSCs cultured for 24‐48 hours in CM of low‐dose OSM (2 ng/ml) also showed significant increases in cell number (Fig. 2B), indicating increased proliferation rates as a result of OSM preconditioning.

Previous studies have shown that both TGF‐β1 and OSM have profibrotic properties and can induce the synthesis of extracellular matrix (ECM) components in fibroblast cells [32, 33, 34]. To assess the antifibrotic effects of preconditioned MSCs, we examined the production of the ECM proteins fibronection and type I collagen in TGF‐β1‐ or OSM‐stimulated MRC‐5 fibroblasts by using an in vitro coculture system. Total collagen content increased in MRC‐5 cells with OSM treatment, but significant collagen content was attenuated under OSM‐MSC coculture conditions (Fig. 2C). TGF‐β1 treatment increased both the fibronectin (Fig. 2D) and type I collagen (Fig. 2E) mRNA expression levels in MRC‐5 cells. As was anticipated, ECM synthesis was significantly attenuated in both the MSC and OSM‐MSC groups, and OSM‐MSCs showed higher inhibitory effects for ECM production than did MSCs (Fig. 2D, 2E). To investigate the anti‐inflammatory effects of MSCs on LPS‐stimulated macrophages, we cocultured RAW264.7 cells with MSC‐conditioned medium simultaneously treated with LPS. LPS treatment increased the level of both pro‐IL1β (Fig. 2F) and IL‐6 (Fig. 2G) mRNA in RAW264.7 cells. Although proinflammatory cytokine was significantly attenuated in both the MSC and OSM‐MSC groups, OSM‐MSCs showed higher inhibitory effects than did MSCs (Fig. 2F, 2G).

**Figure 3:**
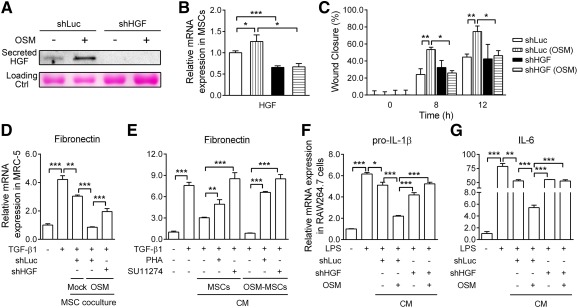
Hepatocyte growth factor secreted in oncostatin M‐preconditioned mesenchymal stem cells attenuates wound healing, extracellular matrix (ECM) production, and inflammatory effects. The HGF‐specific gene is silenced by lentivirus‐mediated shRNA. **(A):** Western blot analysis of the levels of secreted HGF in conditioned medium and **(B)** quantitative real‐time reverse transcription‐polymerase chain reaction (RT‐qPCR) quantification of HGF mRNA levels in cells of the specific HGF knockdown (shHGF‐MSC) under OSM treatments. shLuc‐MSCs are transduced with a scrambled control. Ponceau S staining of the membrane was used as loading control. **(C):** Scratch wound assay of the effect of HGF on MRC‐5 migration. Quantification of wound closure was assessed by TScratch software after 8 and 12 hours. **(D):** Effects of HGF silencing in MSCs on the collagen production. Cocultured OSM‐pretreated shLuc‐ or shHGF‐MSCs (upper chamber) and transforming growth factor‐β1‐treated MRC‐5 (lower chamber) were performed. After 24 hours of incubation, fibronectin mRNA levels were determined. **(E):** Effects of inhibiting HGF signaling with a c‐Met inhibitor on collagen production. MRC‐5 cells were preincubated with PHA‐665752 or SU11274. The cells were then treated with MSCs or OSM‐MSC‐conditioned medium for 24 hours, followed by determination of fibronectin mRNA levels. **(F, G):** Effects of HGF silencing in MSCs on proinflammatory cytokine production, lipopolysaccharides‐treated RAW264.7 cells simultaneously incubated with a mixture of RAW264.7 basal media, and OSM‐pretreated shLuc‐ or shHGF‐MSCs CM or MSCs basal media (1:1). After 7 hours of incubation, RT‐qPCR was performed to examine the pro‐interleukin‐1β and IL‐6 mRNA levels in the cells. Values were normalized to the glyceraldehyde‐3‐phosphate dehydrogenase gene and are expressed in relation to the control group. ∗, *p* < .05; ∗∗, *p* < .01; ∗∗∗, *p* < .001. Abbreviations: CM, conditioned medium; Ctrl, control; h, hour; HGF, hepatocyte growth factor; IL, interleukin; LPS, lipopolysaccharides; MSC, mesenchymal stem cell; OSM, oncostatin M; PHA, PHA‐665752; shHGF, HGF knockdown; TGF, transforming growth factor.

Two approaches were used to clarify whether OSM‐MSCs accelerated wound healing and attenuated ECM production through HGF. First, stable genetic knockdown of HGF with short hairpin RNAs (shRNAs) in MSCs was performed, followed by the use of an in vitro coculture system to assess the wound healing and antifibrotic effects. MSCs transfected with HGF shRNA (shHGF‐MSCs) showed significant decreases in the expression of secreted HGF in the medium in comparison with cells transfected with a control scramble shRNA plasmid (shLuc‐MSCs). Under OSM treatment, HGF showed no significant upregulation in shHGF‐MSCs in comparison with the shLuc‐MSC control group (Fig. 3A). The HGF mRNA level in the cells was further confirmed (Fig. 3B).

Knockdown of HGF also attenuated the accelerated migration effects of OSM‐shLuc‐MSCs on the wound‐healing abilities of MRC‐5 fibroblasts (Fig. 3C). Hence, HGF knockdown also abrogated the suppressive effects of OSM‐shLuc‐MSCs on TGF‐β1‐induced overexpression of fibronectin (Fig. 3D). Second, effects of suppressing HGF signaling by pharmacological inhibition of c‐Met by PHA‐665752 and SU‐11274 were also examined. Results showed that ECM production significantly increased in the MRC‐5 cells treated with TGF‐β1. Although treatment with OSM‐MSC‐conditioned medium clearly suppressed expression of fibronectin, OSM‐MSC CM cotreatment with PHA‐665752 or SU‐11274 significantly inhibited the suppressive effects on fibronectin expression (Fig. 3E). On the other hand, HGF knockdown abrogated the inhibitory effects of OSM‐shLuc‐MSCs on LPS‐stimulated overexpression of pro‐IL1β (Fig. 3F) and IL‐6 (Fig. 3G). The results indicated that paracrine factors, such as HGF, when overexpressed and secreted from OSM‐MSCs, played an important role for the attenuation of ECM production and inflammatory effects.

### OSM‐MSCs Transplantation Attenuates Inflammatory Effects in the Early Stage of BLM‐Induced Pulmonary Fibrosis Mouse Model

**Figure 4:**
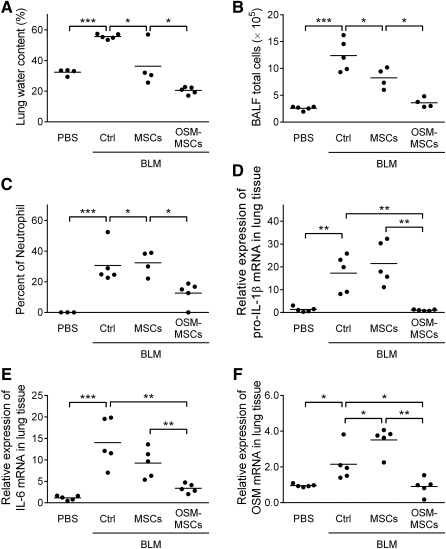
Transplantation of oncostatin M‐preconditioned mesenchymal stem cells attenuates lung edema and bronchoalveolar lavages fluid differential cell counts and downregulates the expression of inflammatory mediators in the early‐stage of a bleomycin‐induced pulmonary fibrosis mouse model. **(A):** Effects of OSM‐MSC treatment on pulmonary edema. The lung water content (in percent) was determined at day 7 post‐BLM treatment. After collecting BALF, the total cells in BALF were counted by a hemocytometer, and cell viability was measured by trypan blue exclusion **(B)** and differential cells in BALF were counted by flow‐cytometric hematology system (XT‐2000iV) **(C)**. Then the lung tissues were collected for inflammatory‐related genes, pro‐interleukin‐1β **(D)**, IL‐6 **(E)**, and OSM mRNA **(F)** expression level detection by quantitative real‐time polymerase chain reaction. Values were normalized to the glyceraldehyde‐3‐phosphate dehydrogenase gene and expressed in relation to the phosphate‐buffered saline group. Each dot represents an individual mouse with the mean shown for *n* ≥ 5 per group. ∗, *p* < .05; ∗∗, *p* < .01; ∗∗∗, *p* < .001. Abbreviations: BALF, bronchoalveolar lavages fluid; BLM, bleomycin; Ctrl, control; IL, interleukin; MSC, mesenchymal stem cell; OSM, oncostatin M; PBS, phosphate‐buffered saline.

PBS, MSCs, or OSM‐MSCs were administered on day 3 after BLM treatment. Mice treated with BLM for 7 days showed a significant increment in the lung water content, indicating the degree of pulmonary edema caused by inflammatory responses in the lung. As anticipated, the BLM‐OSM‐MSCs group showed lower lung water content than the BLM‐MSCs and BLM‐Ctrl groups at day 4 after stem cell transplantation (Fig. 4A). The results also indicated that transplantation of OSM‐MSCs significantly reduced edema caused by BLM treatment. Total cells and neutrophils in BALF were also significantly decreased in BLM‐OSM‐MSCs group on day 4 post‐stem cell transplantation (Fig. 4B, 4C). To assess the effects of OSM‐MSCs on inflammation in the early‐stage of BLM‐induced pulmonary fibrosis model, expression levels of possible inflammatory mediators that may be involved in the process were determined. The mRNA levels of the proinflammatory pro‐IL‐1β, IL‐6, and OSM were significantly upregulated after 7 days of BLM treatment when compared with the PBS group (Fig. 4D–4F). Although MSCs transplantation reduced these inflammatory mediators, there was a more significant reduction in the OSM‐MSCs transplantation group at day 4 post‐stem cell transplantation (Fig. 4D–4F).

### OSM‐MSC Transplantation Improves Pulmonary Respiratory Functions and Downregulates Expression of Fibrotic Factors in the BLM‐Induced Pulmonary Fibrotic Mice

**Figure 5:**
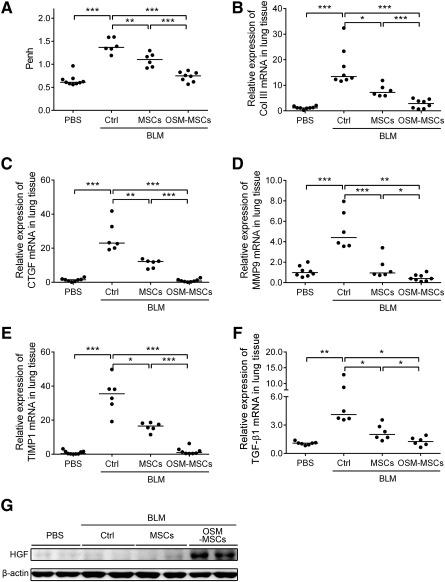
Transplantation of oncostatin M‐preconditioned mesenchymal stem cells improves lung function and downregulates the expression of fibrosis‐related genes in the late‐stage of bleomycin‐induced pulmonary fibrosis mouse model. **(A):** Effects of OSM‐MSCs on pulmonary function. Lung functions were detected as Penh values in animals that received phosphate‐buffered saline (PBS), BLM‐control, BLM‐MSCs, or BLM‐OSM‐MSCs for 18 days following BLM administration. Whole‐body plethysmograph was used, and baseline‐enhanced respiratory pause was used as a noninvasive index of airway dysfunction. Then the lung tissues were collected for fibrotic indicators, Col III **(B)**, connective tissue growth factor **(C)**, matrix metalloproteinase‐9 **(D)**, TIMP1 **(E)**, and transforming growth factor‐β1 **(F)** mRNA expression level detection by quantitative real‐time reverse transcription‐polymerase chain reaction. Values are normalized to the glyceraldehyde‐3‐phosphate dehydrogenase gene and are expressed in relation to the PBS group. Each dot represents an individual mouse with the mean shown for *n* > 5 per group. **(G):** Western blot analysis of HGF in whole lung tissues from each group of mice, treated as described above. ∗, *p* < .05; ∗∗, *p* < .01; ∗∗∗, *p* < .001. Abbreviations: BLM, bleomycin; Col III, collagen III; CTGF, connective tissue growth factor; Ctrl, control; HGF, hepatocyte growth factor; MMP9, matrix metalloproteinase‐9; MSC, mesenchymal stem cell; OSM, oncostatin M; PBS, phosphate‐buffered saline; Penh, baseline‐enhanced respiratory pause; TGF, transforming growth factor; TIMP1, tissue inhibitor of metalloproteinase 1.

To evaluate the therapeutic efficacy of OSM‐preconditioned MSCs in the BLM‐induced pulmonary fibrosis mouse model, we used whole‐body barometric plethysmography to monitor lung functions of the treated mice. The respiratory parameter, in particular the enhanced pause value (Penh), was measured as a noninvasive index of BLM‐induced airway dysfunction. In the experiments, the Penh values showed a 1.5‐fold increment at day 21 after BLM treatment in comparison with the PBS placebo groups (Fig. 5A). In addition, after 18 days of stem cell engraftment (i.e., 21 days post‐BLM treatment), the BLM‐OSM‐MSC groups had the lowest Penh value, which was approximately the same as that of the PBS control; however, the BLM‐MSC group showed only mild improvements in lung functions in the mice (Fig. 5A). We next investigated the effect of OSM‐MSCs on the expression of the main extracellular matrix components, including the major profibrotic growth factor, transforming growth factor‐β1 (TGF‐β1), collagen type III and connective tissue growth factor [35], the matrix metalloproteinase‐9, and the tissue inhibitor of metalloproteinase‐1, all of which specifically contribute to lung fibrosis (Fig. 5B–5F). Expression of these fibrosis‐mediating factors was significantly upregulated at day 21 post‐BLM treatment, but the expression levels were clearly reduced on OSM‐MSC transplantation. Noting the improved therapeutic efficacy, possible cytoprotective mediators were further investigated by examining the expression levels of HGF in whole‐lung tissues by western blot analysis. HGF was overexpressed in the OSM‐MSC‐transplanted mice (Fig. 5G). Taken together, transplantation of OSM‐MSCs showed better inhibitory effects on the expression of the inflammatory and fibrotic mediators and improved lung function in comparison with mice with BLM‐induced pulmonary fibrosis (Figs. 4, 5).

### OSM‐MSC Transplantation Reduces Histological Changes Through HGF Signaling in the Lungs of the BLM‐Induced Pulmonary Fibrotic Mice

**Figure 6:**
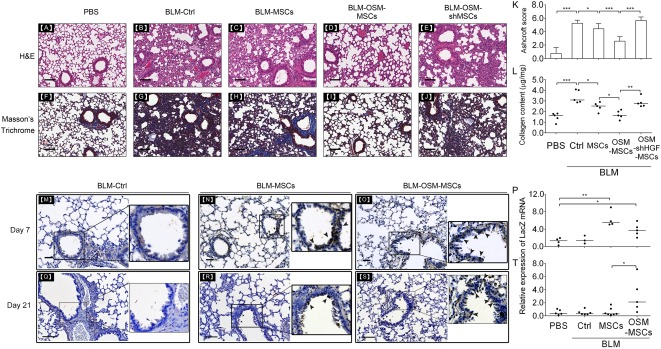
Transplantation of oncostatin M (OSM)‐preconditioned mesenchymal stem cells (MSCs) reduces fibrotic histopathologic changes through hepatocyte growth factor (HGF) signaling in the bleomycin (BLM)‐induced pulmonary fibrosis mouse model. Representative images at day 21 after BLM administration of hematoxylin and eosin **(A‐E)** and Masson’s trichrome‐stained **(F‐J)** histological sections from each group. **(K):** The fibrotic changes in the lung were quantified by using Ashcroft scores, ranging from 0 (normal lung) to 8 (complete fibrosis). **(L):** Total collagen content of whole lung tissues from each group was determined by Sircol Collagen Assay. Immunohistochemistry staining was performed to observe the distribution of LacZ in lung tissues at day 7 **(M‐O)** and day 21 **(Q‐S)** post‐BLM treatments. Quantitative real‐time polymerase chain reaction was performed to detect LacZ expression in the lung tissues of mice that received phosphate‐buffered saline (PBS), BLM‐control, BLM‐MSCs, and BLM‐OSM‐MSCs on day 7 **(P)** and day 21 **(T)** after BLM administration. **(A, F):** PBS control. **(B, G, M, Q):** BLM + PBS. **(C, H, N, R):** BLM + MSCs. **(D, I, O, S):** BLM + OSM‐MSCs. **(E, J):** BLM + OSM‐preconditioned HGF knockdown MSCs. Scale bar, 100 μm. Solid arrows around the bronchi indicate LacZ‐positive cells (brown spots). Values are normalized to the glyceraldehyde‐3‐phosphate dehydrogenase values and expressed in relation to the PBS group. Each dot represents an individual mouse with the mean shown for *n* ≥ 3 per group. ∗, *p* < .05; ∗∗, *p* < .01; ∗∗∗, *p* < .001. Abbreviations: BLM, bleomycin; Ctrl, control; H&E, hematoxylin and eosin; MSC, mesenchymal stem cell; OSM, oncostatin M; PBS, phosphate‐buffered saline; shHGF, HGF knockdown.

The lung histopathologic sections from each experimental group at day 18 post‐stem cell transplantation were examined (Fig. 6). H&E and Masson’s trichrome staining showed that there was no damage or inflammatory infiltration in the lung of the PBS control group (Fig. 6A, 6F), but the pulmonary alveolus cavities of the mice were significantly decreased in size, and the alveolar wall was thickened in the BLM control group (Fig. 6B, 6G). Although lung tissues from the MSC‐transplanted mice showed an obviously thinner alveolar wall (Fig. 6C, 6H), the OSM‐MSC‐transplanted group showed an even more significant reduction of the alveolar wall thickness and accumulation of ECM in the lung interstitium (Fig. 6D, 6I). However, the BLM‐OSM‐shHGF‐MSC‐transplanted group obviously abrogated the antifibrotic effect of the BLM‐OSM‐MSC group (Fig. 6E, 6J). Quantitative histologic analysis by measuring the Ashcroft score showed that OSM‐MSC transplantation significantly decreased the fibrotic effects in the BLM injury model (Fig. 6K). In addition, there was significant collagen accumulation after BLM administration, but BLM‐OSM‐MSC groups showed higher inhibitory effects for collagen deposition than did BLM‐MSC groups (Fig. 6L). The results also indicated that HGF overexpressed in the OSM‐MSCs played an important role for the attenuation of fibrosis. To determine whether the engrafted OSM‐MSCs adapted to the microenvironment in the damaged lungs at days 4 and 18 post‐stem cell engraftment, we intratracheally administered LacZ reporter‐transduced MSCs (β‐Gal‐MSCs) into the BLM‐treated mice. Immunohistochemical staining showed no LacZ‐labeled cells or LacZ mRNA in lung tissue sections of the BLM control group at day 7 (Fig. 6M, 6P) and day 21 (Fig. 6Q, 6T) after BLM treatment. RT‐qPCR showed approximately a sixfold increment of LacZ mRNA level, and numerous LacZ‐labeled cells were observed to be distributed around the bronchi in both the BLM‐MSC and BLM‐OSM‐MSC groups at day 4 post‐stem cell engraftment (Fig. 6N‐6P). At day 18 posttransplantation, approximately a twofold increment of LacZ mRNA as well as numerous LacZ‐labeled cells were observed that were distributed around the bronchi in the BLM‐OSM‐MSC groups (Fig. 6S, 6T). However, there were only a few LacZ‐labeled cells or LacZ mRNA observed in the MSC‐transplanted group at day 18 after stem cell transplantation (Fig. 6R, 6T).

Taken together, our data indicated that oncostatin M preconditioning enhanced the survival rate of engrafted MSCs, exerted better suppression effects on inflammation and fibrosis, and improved lung functions in BLM‐induced pulmonary fibrotic mice.

## Discussion

OSM exerts multiple functions, such as regulation of stem cell pluripotency, induction of osteogenesis in MSCs [36], and modulation of differentiation of hepatocytes from human embryonic stem cells [37], MSCs [38], or pluripotent stem cells [39]. Previous studies have reported that OSM treatment increases gp130 promoter activities and enhances the synthesis of OSMRβ and gp130, and to a lesser extent, LIFR in various cell lines [40]. Our data, indeed, showed that OSM preconditioning specifically upregulated type 2 OSM receptors (Fig. 1A, 1D) rather than the IL‐6 family‐related receptors, IL‐6R and LIFR (supplemental online Fig. 1A).

In addition, OSM also exhibits diverse biological activities such as regulation of inflammation, remodeling of ECM, wound healing, and modulation of cell growth in a wide variety of cells in both in vivo and in vitro studies [41]. Data presented in this work are consistent with these findings. Depending on the targeted cell types, OSM may reduce growth of melanoma, breast cancer, and glioma cells, as well as normal and neoplastic mammary epithelial cells [31]. On the other hand, OSM has been shown to promote proliferation of trophoblasts, intestinal epithelial cells, and human adipose tissue‐derived MSCs [17, 28]. This report adds to the expanding list showing that OSM also stimulates proliferation of bone marrow MSCs (Fig. 2B).

Previous studies have described OSM stimulation of the production of extracellular matrix components in human lung fibroblasts [33]. Elevated levels of OSM have been shown to induce ECM remodeling in BLM‐induced pulmonary fibrosis animal models [42] and in clinical studies [43]. In our study, the OSM mRNA level was significantly upregulated in lung tissues of the early stage of BLM‐induced lung fibrosis mouse model (Fig. 4F). Furthermore, OSM stimulated the production of collagen in MRC‐5 fibroblast (Fig. 2C), suggesting a role for OSM in pulmonary fibrosis. On the other hand, OSM administration attenuated oxidative stress‐induced cardiac ischemia/reperfusion injury by inhibiting cardiomyocyte apoptosis through upregulating Bcl‐2 expression [44]. In contrast, another study showed that OSM treatment may enhance the H_2_O_2_‐induced cell apoptosis [45]. However, OSM‐MSCs in this study showed no significant protection against H_2_O_2_‐induced cell death when compared with MSCs (supplemental online Fig. 1B). Then, determination of the antiapoptotic gene, Bcl‐2, mRNA expression levels showed significant downregulation in different OSM‐pretreated MSCs. In addition, the expression of the antioxidant genes, *heme oxygenase 1 (*HO‐1) and catalase, were also detected. The expression level of both genes clearly downregulated in the OSM‐pretreated MSCs; only 2 ng/ml of OSM‐treated MSCs showed slightly increased expression of HO‐1 (supplemental online Fig. 1C). Thus, OSM‐preconditioned MSCs showed no improvement on oxidative stress‐induced cell death.

Overproduction of ECM and imbalance of MMPs and TIMPs are the hallmark of fibrosis. Mounting evidence highlights the therapeutic potentials for the transplantation of MSCs or extracellular vesicles in lung fibrosis models to act simultaneously on immune responses [46] and fibrogenesis mechanisms [47, 48]. On the other hand, transplanted MSCs may suppress lung fibrosis and inflammation through downregulating expression of MMPs [49] and TIMPs in lung fibrosis models [50]. Consistent with other reports, our study also shows that OSM‐MSCs possessed better cytoprotection and attenuated stimuli‐mediated inflammatory response and ECM production in vitro and in vivo.

Accumulating evidence has shown that MSCs act as guardians against excessive inflammation via secretion of paracrine factors that modulate inflammatory responses [51]. MSCs engrafted to an endotoxin‐induced lung injury model significantly attenuated pulmonary proinflammatory mediators while increasing the anti‐inflammatory cytokine IL‐10 [52]. MSC therapy inhibits the progression of BLM‐induced pulmonary fibrosis by altering proinflammatory cytokine tumor necrosis factor‐α and IL‐1β [53, 54] and by reducing the infiltration of inflammatory cells, such as neutrophils and lymphocytes [50], in a paracrine way. Administration of MSC‐derived conditioned medium reduced the number of inflammatory cells and proinflammatory cytokine production within the BALF in hyperoxia‐induced lung injuries [55]. Intrapulmonary administration of MSCs was shown to ameliorate cigarette smoke‐induced emphysema in part via downregulation of proinflammatory mediators [56]. Hence, OSM‐MSC transplantation exerts a better suppressive effect on inflammatory cell infiltration (Fig. 4B, 4C) and proinflammatory cytokine production in the early stage of BLM‐induced lung fibrotic animals (Fig. 4D–4F).

Xu et al. showed that transplantation of preconditioned cardiac cells or MSCs via the activation of angiotensin II type 2 receptor produced a better outcome of heart function and reduced infarct size [57]. Others found that MSCs overexpressing toll‐like receptor 3 secreted more multifunctional trophic factors to inhibit killing of natural killer cells and enhanced therapeutic potency [58, 59]. On the other hand, Wharton’s Jelly‐derived MSCs highly expressing TβRI/ALK5 and TβRII exhibited a higher susceptibility to TGF‐β1 and secreted paracrine factors for intervertebral disc regeneration with enhanced regenerative efficacy [60]. In this work, MSCs overexpressing the type 2 OSM receptor showed a better response to OSM, prolonged survival of the engrafted cells, and improved the therapeutic effects (Fig. 6) by secretion of the paracrine factor HGF (Figs. 5G, 6E, 6J–6L).

Recent studies have demonstrated that HGF secreted by MSCs exerts multiple protective effects for preventing fibrosis [61]. HGF plays a key role in modulating inflammatory response [62] and wound repair ability [63], in attenuating stimuli‐induced epithelial mesenchymal transition and apoptosis in type II alveolar epithelial cells [61], and in inhibiting myofibroblast differentiation and ECM production [64]. In agreement with this reported evidence, our results showed that HGF was upregulated and secreted from OSM‐MSCs that attenuated stimuli‐induced inflammatory response and ECM production and promoted normal lung fibroblast wound repair in vitro. In addition, HGF was significantly upregulated in the OSM‐MSC‐treated fibrotic lungs and attenuated BLM‐induced lung injury and fibrosis in vivo in the present study (Fig. 5G).

## Conclusion

In conclusion, we report here that preconditioning MSCs with the cytokine OSM prolonged the survival of engrafted cells in BLM‐induced fibrotic lungs, and significantly enhanced the sensitivity to activate cytoprotecive factors, possibly HGF, downstream of OSM. Such events resulted in improvement in pulmonary functions and in attenuation of inflammatory and fibrotic mediators. Our data strongly suggest that preconditioning MSCs with OSM prior to transplantation is a better therapeutic strategy for treating lung fibrosis.

## Author Contributions

Y.‐W.L.: collection and/or assembly of data, data analysis and interpretation; animal handling; S.‐M.T.: collection and/or assembly of data, data analysis and interpretation; T.‐T.H.: collection and/or assembly of data, animal handling; K.‐B.C. and H.‐P.K.: conception and design, manuscript writing; C.‐M.C.: conception and design, data analysis and interpretation, financial support; K.‐Y.C.: conception and design, data analysis and interpretation, financial support, final approval of manuscript.

## Disclosure of Potential Conflicts of Interest

The authors indicated no potential conflicts of interest.

## Supporting information

Supporting Information
