# Supplementary material for: Oncostatin M‐Preconditioned Mesenchymal Stem Cells Alleviate Bleomycin‐Induced Pulmonary Fibrosis Through Paracrine Effects of the Hepatocyte Growth Factor
Source: Stem Cells Transl Med. 2016 Oct 18;6(3):1006–17. doi: 10.5966/sctm.2016-0054 (PMC5442768; doi:10.5966/sctm.2016-0054)
Supplement: Supplementary file 1 — Supporting Information [file SCT3-6-1006-s001.pdf]

Supplemental Information – Chong et al.

Supporting Information Figure

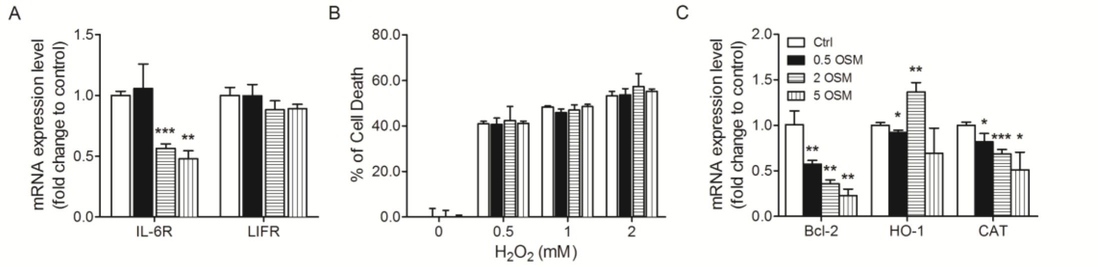

Figure S1. Effects of oncostatin M preconditioning on IL-6 family-related receptors complex and expression of cytoprotective genes in MSCs. Real-time RT-PCR quantification of the relative expression levels of mRNAs of IL-6 family-related receptor (A) IL-6R and LIFR in MSCs treated with different dosages of OSM for 24 h. (B) Cell viability of MSCs and OSM-MSCs treated with the indicated H_2O_2 concentration for 1 h as assessed by MTT analysis. (C) mRNA levels of anti-apoptotic factors, Bcl-2, and anti-oxidant genes, CAT and HO-1, in MSCs as determined by real-time RT-PCR. Values were normalized to GAPDH and are expressed relative to the respective control group. *p<0.05, **p<0.01, and ***p<0.001.

Supporting Information Table

Table S1. Primer Sequences

Gene	Primer Sequence
OSMR	Forward: AAGGTTTTCCACGGTGAGTG
	Reverse: TTAAACCAGGCGTCCGTTAC
gp130	Forward: TGCACAGTGAAGGAAGTTCG
	Reverse: AGTCTGGGTGGAAGCAGAGA
HGF	Forward: GGCTGAAAAGATTGGATCAG
	Reverse: AGGAACAATGACACCAAGAACCA
Bcl-2	Forward: TACCGTCGTGACTTCGCAGAG
	Reverse: CAGGCTGAGCAGGGTCTT
CAT	Forward: CCTGACATGGTCTGGGACTT
	Reverse: CAAGTTTTTGATGCCCTGGT
HGF	Forward: GGCTGAAAAGATTGGATCAG
	Reverse: TGGTTCTTGGTGTCATTGTTCCT
HO-1	Forward: AAGCCGAGAATGCTGAGTTCA
	Reverse: GCCGTGTAGATATGGTACAAGGA
Fibronectin	Forward: CCCACCGTCTCAACATGCTTAG
	Reverse: CTCGGCTTCCTCCATAACAAGTAC
OSM	Forward: TCCGCCTCCAAAACCTGAACAC
	Reverse: ATGGTATCCCCAGAGAAAGC
Pro-IL-1β	Forward: GCTCATCTGGGATCCTCTCC
	Reverse: CCTGCCTGAAGCTCTTGTTG
IL-6	Forward: CCACTTCACAAGTCGGAGGCTTA
	Reverse: GCAAGTGCATCATCGTTGTTCATAC
Col III	Forward: GTTCTAGAGGATGGCTGTACTAAACACA
	Reverse: TTGCCTTGCGTGTTTGATATTC
CTGF	Forward: ACCTGGAGGAAAACATTAAGAAGG
	Reverse: AGCCCTGTATGTCTTCACACTG
MMP9	Forward: CTGGACAGCCAGACACTAAAG
	Reverse: CTCGCGGCAAGTCTTCAGAG
TIMP1	Forward: GCAACTCGGACCTGGTCATAA
	Reverse:CGGCCCGTGATGAGAAACT

LacZ	Forward: CCGTTGATGTTGAAGTGGC	
	Reverse: CTAATCCGAGCCAGTTTACCC	
IL6R	Forward: CCGTGAACTCCTTTGACCAT	
	Reverse: GAGATCCTGGAGGGTGACAA	
LIFR	Forward: ACACGACCACATGGTTCTCA	
	Reverse: GCATGTTTGTGGTGACCAAG	
TGF-β1	Forward: TTGCTTCAGCTCCACAGAGA	
	Reverse: TGGTTGTAGAGGGCAAGGAC	
β-Actin	Forward:GCGAGAAGATGACCCAGATC	
	Reverse:CCAGTGGTACGGCCAGAGG	
GAPDH	Forward: AGAGACGGCC GCATCTTCTT	
	Reverse: CCGTTCACACCGACCTTCAC	

OSMR, oncostatin M receptor; gp130, glycoprotein 130; HGF, hepatocyte growth factor; HO-1, heme oxygenase-1*; Bcl-2,* B-cell lymphoma 2; CAT, catalase; Col III, collagen type III; CTGF, connective tissue growth factor; MMP9, matrix metallopeptidase 9; TIMP1, tissue inhibitor of metalloproteinase 1; IL6R, interleukin 6 receptor; LIFR, leukemia inhibitory factor receptor; TGF-β1, transforming growth factor beta-1.
